# A prospective study of nutrition education and oral nutritional supplementation in patients with Alzheimer's disease

**DOI:** 10.1186/1475-2891-10-98

**Published:** 2011-09-26

**Authors:** Glaucia AK Pivi, Rosimeire V da Silva, Yara Juliano, Neil F Novo, Ivan H Okamoto, César Q Brant, Paulo HF Bertolucci

**Affiliations:** 1 Department Neurology and Neurosurgery, Behaviour Neurology Section, Universidade Federal de São Paulo/UNIFESP-EPM, São Paulo, Brazil; 2 Department of Nutrition, Universidade de Santo Amaro/UNISA, São Paulo, Brazil

**Keywords:** supplementation, nutritional education, Alzheimer's disease

## Abstract

**Background:**

Weight loss in patients with Alzheimer's disease (AD) is a common clinical manifestation that may have clinical significance.

**Objectives:**

To evaluate if there is a difference between nutrition education and oral nutritional supplementation on nutritional status in patients with AD.

**Methods:**

A randomized, prospective 6-month study which enrolled 90 subjects with probable AD aged 65 years or older divided into 3 groups: Control Group (CG) [n = 27], Education Group (EG) [n = 25], which participated in an education program and Supplementation Group (SG) [n = 26], which received two daily servings of oral nutritional supplementation. Subjects were assessed for anthropometric data (weight, height, BMI, TSF, AC and AMC), biochemical data (total protein, albumin, and total lymphocyte count), CDR (Clinical Dementia Rating), MMSE (Mini-mental state examination), as well as dependence during meals.

**Results:**

The SG showed a significant improvement in the following anthropometric measurements: weight (H calc = 22.12, p =< 0.001), BMI (H calc = 22.12, p =< 0.001), AC (H calc = 12.99, p =< 0.002), and AMC (H calc = 8.67, p =< 0.013) compared to the CG and EG. BMI of the EG was significantly greater compared to the CG. There were significant changes in total protein (H calc = 6.17, p =< 0.046), and total lymphocyte count in the SG compared to the other groups (H cal = 7.94, p = 0.019).

**Conclusion:**

Oral nutritional supplementation is more effective compared to nutrition education in improving nutritional status.

## Background

Weight loss in patients with Alzheimer's disease (AD) is a common clinical manifestation. In these patients, impaired nutritional status results in changes in body composition and biochemistry indicators [1]. In order to identify such weight loss, some studies correlated organic deficiency and low energy intake or hypercatabolism in patients, thus suggesting that weight loss may be a risk factor in the etiology of dementias and other psychiatric and cognitive disorders, however this has not been evaluated [2-4]. Higher infection rates, increased energy expenditure due to repetitive movements, and cognitive deficit impairing AD patient independence may also be considered as a cause of weight loss [5,6]. Weight loss increases the risk for infections, pressure ulcer development and poor wound healing, which in turn can impair AD patient's quality of life [7].

Some strategies can be adopted to improve the nutritional status of these patients. These strategies include patient nutrition education programs, and the use of oral nutritional supplements, which can significantly impact nutritional status [8-10]. Thus, the need to study strategies of nutritional intervention which minimize or improve AD patients' nutritional status is justified, supporting the conduction of this study. The objective of this study is to determine if there is any difference between oral nutritional supplementation and nutrition education on the nutritional status of patients with AD.

## Methodology

A randomized, 6-month, prospective study was conducted at the clinic of the Behavioral Neurology Sector, in the Neurology and Neurosurgery Department of Universidade Federal de São Paulo - Escola Paulista de Medicina (UNIFESP/EPM).

The sample consisted of 90 subjects, of both genders, aged at least 65 years old and with probable AD, according to Diagnostic Statistical Manual 4^th ^Edition (DSM IV) criteria and clinical dementia rating (CDR) of 1, 2 or 3.

For exclusion criteria were considered other forms of dementia, alternative feeding requirement (tube feeding), type 1 and 2 diabetes mellitus, and renal diseases.

Twelve subjects were included in the study but not in the statistical analys is: 3 subjects from CG and 4 from EG had difficulty in being transported to the hospital; 3 subjects from SG and 1 from CG died; 1 subject from SG needed tube feeding.

The remaining subjects were randomized into 3 groups: control group (CG), (N = 27), education group (EG), (N = 25), and supplementation group (SG), (N = 26).

All subjects were assessed at baseline and then at monthly intervals during the 6-month study period, including an orientation of health nutrition. The subjects nutritional status was assessed using anthropometric and biochemical data. The anthropometric data included: height (m), current weight (kg), Body Mass Index (BMI) (kg/m^2^), arm circumference (AC) (cm), arm muscle circumference (AMC) (cm), and triceps skinfold (TSF) (mm). For weight were used Welmy^® ^mechanical scales for adults with a 150 kg capacity and for height was utilized stadiometer graduated in centimeters, to for subjects that were able to maintain erect posture. For the others with presented posture problems as kyphosis or lordosis and were usable to stand, the stature and knee equation proposed by Chumlea (1985) [11] was used, to avoid biases in measures of statures. For the compartmental muscle and fat mass assessment, Lange^® ^Skinfold calipers were used, which expresses results in millimeters (mm). The biochemistry data included: total protein (TP), serum albumin and total lymphocyte count (TLC). Biochemical data were collected following a 12-hour fast and evaluated by the central laboratory of Hospital São Paulo.

For cognitive tracking, all subjects were assessed using the Mini-Mental State Examination (MMSE) assessing temporal and spatial orientation, memory, attention, calculation, language and praxis [12,13] and Clinical Dementia Rating (CDR) [14,15] administered by a duly qualified neuropsychologist.

The following demographic data were also collected for all subjects: education level (years of schooling), time of disease evolution calculated from the date of diagnosis and dependence during meals.

Subjects in the CG were monitored by monthly nutritional assessments and did not receive any form of intervention. The 25 Subjects caregivers and patients, in the EG participated in the educational program which consisted of 10 classes. Each class was taught to a maximum of 10 participants, with the aim greater interaction between the professional and caregivers. Each expositive class was supported by slides, with themes were of data proposed in accordance with the Brazilian Association of Alzheimer's (ABRAZ) Caovilla & Canineu (2002) [16] a nonprofit association that assists caregivers and family members of patients with Alzheimer's disease. Also it took into account the information received from the caregivers based on the main nutrition deficits in AD patients. The classes were developed with relevant topics to the needs of nutritional intervention, such as: the importance of nutrition in disease, behavioral changes during meals, attractive meals, constipation, hydration, administration of drugs, swallowing, food supplementation, lack of appetite, clarification of doubts. This order of exposition follows the progression of AD and the onset of symptoms that may be related to nutrition. Subjects in the SG received oral nutritional supplementation twice daily for 6 months in addition to their usual diet (Ensure with FOS^®^, Abbott Nutrition) and were measured monthly by means of anthropometry and biochemical parameters, as described before. Two servings provide 680 kcal and 25.6 g protein/day.

All subjects or their representatives signed the Informed Consent Form. This research was approved by the Ethics Committee of Universidade Federal de São Paulo (protocol 0552/06).

Statistical analysis was undertook using Kruskal-Wallis variance analysis to compare the three groups in relation to anthropometric, biochemistry, demographic and disease stage variables, and Siegel's chi-square test to study possible associations between the groups and the variables studied. The rejection level of the null hypothesis was fixed at α = 0.05.

## Results

Ninety (90) subjects were enrolled in the study, and 86.67% (n = 78) completed the study. Of the subjects, 30% (n = 27) were in the CG; 27.78% (n = 25) were in the EG; and 28.88% (n = 26) were in the SG. 8.89% (n = 8) of the patients did not meet the inclusion criteria and 4.44% (n = 4) died.

Of the 78 subjects who completed the study, 67.9% (n = 53) were female and 32.1% (n = 25) were male, with mean age of 75.2 years and median age of 76 years.

As shown in tables 1 and 2, 39.7% (n = 31) were in the moderate phase of the disease (CDR 2) and had a mean MMSE of 12.32, however there were no statistically significant differences among the groups. Demographic data (table 1) as well as dependence during meals (table 2) were also not statistically different among the groups. All these variables show that the groups were similar and did not have any influence on the results obtained.

**Table 1:** Demographic and MMSE data of Alzheimer's Disease (AD) patients, according to Control Group (CG), Education Group (EG) and Supplementation Group (SG)

	Control		Education		Supplementation		Kruskal-Wallis variance analysis		
	Mean	Median	Mean	Median	Mean	Median	Calculated H	*P*	Significance
MMSE	12.59	18.00	12.80	12.00	11.58	9.00	0.14	0.932	N.S.
Age	75.22	74.00	75.88	76.00	76.38	78.00	0.61	0.738	N.S.
School.	3.44	3.00	5.40	4.00	4.61	4.00	3.62	0.164	N.S.
Tempev.	5.93	5.00	6.40	6.00	6.19	6.00	0.45	0.798	N.S.

MMSE: mini-mental state examination; School.: schooling in years; TEMPEV.: time of disease evolution; *p*: significance level; N.S.: not significant.

**Table 2:** Patients with Alzheimer's Disease (AD) in Control Group (CG), Education Group (EG) and Supplementation Group (SG) compared for CDR, gender and dependence during meals

	CDR			Gender		Dependence during Meals	
	
	1 (n)	2 (n)	3 (n)	Male (n)	Female (n)	Not dep.	Dep.
CG	9	9	9	7	20	14	13
EG	7	11	7	8	17	17	8
SG	7	11	8	10	16	13	13
Total	23	31	24	25	53	44	34

Critical *X*^*2 *^= 5.99 calculated *X*^*2 *^for CDR = 0.77 not significant

Critical *X*^*2 *^= 5.99 calculated *X*^*2 *^for gender = 0.96 not significant

Critical *X*^*2 *^= 5.99 calculated *X*^*2 *^for dependence during meals = 2.03 not significant

Regarding the anthropometric and biochemical parameters (Table 3), the SG showed a significant improvement in the anthropometric data of weight (H calc = 22.12, p =< 0.001), body mass index (H calc = 22.12, p =< 0.001), arm circumference (H calc = 12.99, p = 0.002), and arm muscle circumference (H calc = 8.67, p = 0.013) compared to CG and EG after 6 months. There were no statistically significant differences in TSF among the three groups after 6 months. The BMI in the EG was significant compared to CG. There were significant differences in total protein (H calc = 6.17, p = 0.046) in the SG compared to the other groups. The total lymphocyte count in the SG and EG was significant compared to the CG (H cal = 7.94, p = 0.019) after 6 months.

**Table 3:** Mean and median values of anthropometric and biochemical measures for patients with Alzheimer's disease in the different groups

	Control		Education		Supplementation		Kruskal-Wallis		
	Mean	Median	Mean	Median	Mean	Median	Calculated H	*p*	Significance
Height	1.57	1.57	1.54	1.54	1.58	1.56	02.20	0.333	N.S.
CW	-2.20 (61.87 - 60.65)	-1.81 (57.60 - 55.70)	1.19 (54.29 - 50.00)	0.32 (54.54 - 51.70)	6.66 (54.70 - 57.93)	4.37 (53.55 - 56.80)	22.12	<0.001	S > C and E
BMI	-2.21 (24.81 - 24.32)	-1.82 (23.55 - 22.80)	1.19 (22.71 - 22.84)	0.31 (23.82 - 23.90)	6.55 (21.66 - 22.98)	4.37 (22.38 - 23.15)	21.94	<0.001	S > C and E
AC	-0.41 (26.14 - 26.07)	0.00 (26.40 - 26.30)	1.87 (24.72 - 25.11)	2.34 (25.60 - 26.00)	5.44 (23.99 - 25.20)	4.76 (23.90 - 24.80)	12.99	0.002	S > C and E
TSF	2.20 (15.67 - 15.85)	0.00 (15.00 - 16.00)	2.32 (14.20 - 16.40)	1.00 (14.00 - 16.00)	1.44 (11.54 - 13.55)	1.58 (12.00 - 14.00)	3.98	0.136	N.S.
AMC	-0.19 (21.21 - 21.60)	-0.90 (21.12 - 21.01)	-1.27 (20.25 - 19.96)	-2.06 (19.80 - 19.66)	3.43 (20.36 - 21.01)	2.54 (20.44 - 20.93)	8.67	0.013	S > C and E
Total prot.	0.09 (06.94 - 06.95)	0.00 (07.00 - 06.90)	-1.04 (06.55 - 06.84)	-1.39 (06.80 - 06.80)	4.30 (06.46 - 07.02)	3.28 (06.8 - 07.00)	6.17	0.046	S > C and E
Albumin	-3.15 (04.31 - 04.17)	-4.35 (04.30 - 04.10)	-4.06 (04.05 - 04.08)	-4.54 (04.20 - 04.20)	0.69 (03.81 - 04.07)	0.00 (04.20 - 04.20)	2.54	0.281	N.S.
TLC	13.43 (2045.24 - 2279.46)	10.50 (2034.35 - 2416.04)	-1.06 (1888.85 - 1835.25)	-1.41 (1852.40 - 1806.12)	12.57 (1464.20 - 1808.68)	10.68 (1541.10 - 1582.50)	7.94	0.019	S and E > C

CW = Current Weight (kg); BMI = Body Mass Index (Kg/m^2^); AC = Arm Circumference (cm); TSF = Triceps Skin fold (mm) and AMC = Arm Muscle Circumference (cm); Total prot. = total serum protein (μg/dl); TLC = Total Lymphocyte Count (mm^3^); *p*: significance level; N.S. = not significant

There were no statistically significant differences in serum albumin among the three groups.

## Discussion

Most subjects studied had CDR 2, which is relevant since most feeding behavior changes, such as forgetfulness or feeding voracity, are seen in this phase of the disease [17].

In the EG, BMI showed a significant increase compared to CG (p < 0.001). Likewise, biochemical (TP and TLC, p = 0.046, p = 0.019 respectively) and anthropometric indicators (AC and AMC, p = 0.002 and p = 0.013 respectively) also improved in EG group. Our results support the findings from other studies, showing that nutritional education has a positive effect on the diet and modifies nutrition knowledge [18,7,19].

There were significant differences in the SG's anthropometric measures (current weight, BMI, AC, AMC) compared to CG and EG, showing that the use of oral nutritional supplementation improves the patient's nutritional status. In addition, since there were no significant differences in TSF measurement among the three groups, and particularly in the SG, showed that there was no increase in subcutaneous body fat. Furthermore, the oral nutritional supplementation did not result in significant difference in TSF measurement among the three groups, indicating that additional oral nutritional supplementation did not increase subcutaneous body fat in this sample.

Oral nutritional supplementation provided to dementia patients significantly improves nutritional status and the quality of the diet consumed [20,21], but despite these results, Trelis & López [22] observed that only 11% of outpatients used oral nutritional supplements.

In Brazil, there are no studies of outpatients using any type of oral nutritional supplements. However, this study clearly indicates that it is important for all health care professionals involved in the treatment of patients with AD to be able to detect the presence of nutritional deficits. This enables health care professionals to refer the patient to a qualified health professional, so the best nutritional intervention can be implemented.

The benefits of using oral nutritional supplementation have also been studied in elderly subjects without dementia and have shown their efficacy in addition to usual diet. The addition of 500 kcal/day via oral nutritional supplementation for elderly has been shown to improve convalescence and recovery from deficiency states [23,24].

In relation to the biochemical parameters used to assess nutritional status, significant differences were only seen for total protein in the SG and total lymphocyte count in the SG and EG.

Increased total protein in the SG was not correlated with the use of oral nutritional supplementation, but this result may be correlated with evolution of the disease itself. This parameter could perhaps be better assessed with a larger sample or longer follow up.

Increased total lymphocyte count (TLC) in the EG suggests that nutrition education might contribute to the improvement in the immune status of the patients. This may be due to the influence of education on the choice of healthier food, which contributes to the nutritional status as a whole [25].

On the other hand, the significant improvement in TLC in SG subjects showed that the use of oral nutritional supplementation improves immune status. This may be due to the antioxidant micronutrients in the supplement, particularly selenium, β-carotene, vitamin C and E, which impact with the immune system [26,27].

Improving or maintaining the nutritional status of patients with AD should be a priority of patient treatment. Therefore this is a responsibility for the entire multiprofessional team.

Implementing nutrition education improved the BMI of patients with AD, and thus its use is viable for any kind of healthcare service due to its low cost and positive impact on the modification of dietary habits. In addition, oral nutritional supplementation should be part of the usual diet of these patients, as the additional nutrients provided contribute to an improved nutritional status [28,29].

## Conclusion

Oral nutritional supplementation was shown to be more effective compared to nutrition education in improving nutritional status of patients with Alzheimer's Disease.

## List of abbreviations

ABRAZ: Brazilian Association of Alzheimer's; AC: Arm Circumference; AD: Alzheimer's Disease; AMC: Arm muscle circumference; BMI: Body Mass Index; CDR: Clinical Dementia Rating; CG: Control Group; DSM IV: Diagnostic Statistical Manual 4^th ^Edition; EG: Education Group; FOS: Fructooligosaccharides; g: gram; Kcal: calorie; Kg: kilogram; Kg/m2: kilogram/meter; mm: millimeters; MMSE: Mini-Mental State Examination; SG: Supplementation Group; TLC: Total Lymphocyte Count; TP: Total protein; TSF: Triceps skinfold;

## Competing interests

The authors declare that they have no competing interests.

## Authors' contributions

GP gave classes, nutritional orientations and antropometric measures in all sample, writing of the article and critically reviewed the article. RS participated of protocol desing, did cognitive measures and reviewed the manuscript. YJ and NN carried out of the statistical analysis. IO was responsible for DA diagnostics. CQ and PH was involved in the protocol and the study desing, analysis and writing of the article. All authors read and approved the final manuscript.
